# The impact of preoperative treatments on the immune environment of rectal cancer

**DOI:** 10.1111/apm.13467

**Published:** 2024-09-10

**Authors:** Erkki‐Ville Wirta, Hanna Elomaa, Maarit Ahtiainen, Marja Hyöty, Toni T. Seppälä, Teijo Kuopio, Jan Böhm, Jukka‐Pekka Mecklin, Juha P. Väyrynen

**Affiliations:** ^1^ Department of Gastroenterology and Alimentary Tract Surgery Tampere University Hospital Tampere Finland; ^2^ Faculty of Medicine and Health Technology, Tampere University and Tays Cancer Center Tampere University Hospital Tampere Finland; ^3^ Department of Biological and Environmental Science University of Jyväskylä Jyväskylä Finland; ^4^ Department of Education and Research The Wellbeing Services of Central Finland Jyväskylä Finland; ^5^ Department of Pathology Wellbeing Services County of Central Finland Jyväskylä Finland; ^6^ Department of Gastrointestinal Surgery Helsinki University Central Hospital, University of Helsinki Helsinki Finland; ^7^ Applied Tumor Genomics, Research Program Unit University of Helsinki Helsinki Finland; ^8^ Faculty of Sport and Health Sciences University of Jyväskylä Jyväskylä Finland; ^9^ Translational Medicine Research Unit, Medical Research Center Oulu Oulu University Hospital and University of Oulu Oulu Finland

**Keywords:** Tumor‐infiltrating lymphocytes, Crohn's‐like reaction, short‐course radiotherapy, chemoradiotherapy, tumor regression

## Abstract

To improve local disease control, the use of preoperative radiotherapy either alone or combined with chemotherapy has become standard practice in rectal cancer, but it is unclear how these treatments modify the antitumoral immune response. We aimed to evaluate tumor histopathologic features and the prognostic effect of host immune response in rectal cancer with variable treatment modalities. Ninety‐five rectal cancers with short‐course radiotherapy (SRT), 97 with long‐course chemoradiotherapy (CRT), and 154 without preoperative treatments, were evaluated for histopathologic features including Crohn's‐like reaction (CLR). CD3+ and CD8+ immunohistochemistry and tumor cells were analyzed from tumor tissue microarray samples to calculate T‐cell densities and G‐cross function values to estimate cancer cell–T‐cell co‐localization (proximity score). We found that lymphocyte densities were diminished after SRT, but CLR was scarcer after CRT. Proximity score and CLR density were prognostic for survival in cancer without preoperative treatments and could be combined into an enhanced prognostic score (immune grade). In the irradiated tumors, CLR density remained prognostic while the impact of T‐cell infiltration was insufficient alone. In multivariable analysis, the immune grade proved to be an independent prognostic factor for survival. In conclusion, the immune contexture of rectal cancer harbors prognostic significance even after preoperative radiotherapy.

Rectal cancer, although often referred to as part of colorectal cancer (CRC), has many unique features compared to colon cancer. The rectum gives rise to about one third of all CRCs, one of the most significant malignancies worldwide [1]. The partly extraperitoneal anatomic location in the small pelvis is surgically challenging, considering the limited space with adjacent pelvic vessels, nerves, and urinary and sexual organs [2]. In this regard, rectal cancer surgery is not only associated with high morbidity but also with a high local recurrence rate. The surgery first approach for Stages II–III cancer originally had a very high local recurrence rate. The appropriate surgical technique with total mesorectal excision (TME) reduces this risk, and the results for the early‐stage disease are excellent, but the local recurrence rate for higher risk Stage III tumors without preoperative therapy remains at 20–30% [3, 4].

To further improve the locally advanced disease control, the use of preoperative radiotherapy either alone or combined with chemotherapy has become standard practice in rectal cancer. The currently recommended preoperative treatments for locally advanced cancer are either short‐course radiotherapy (SRT) of 25 Gy for 1 week (5 × 5 Gy), followed by surgery usually within 10 days of the first radiation fraction, or long‐course chemoradiotherapy (CRT) of 45–50 Gy in 25–28 fractions typically combined with a fluoropyrimidine‐based radiosensitizer, followed by surgery within 8–10 weeks of the last radiation fraction. CRT is advised when the circumferential margin is threatened according to the radiological assessment or there is a need for tumor regression to ensure an R0 resection [5]. Neoadjuvant CRT considerably improves the local control rate of the advanced disease, while this does not seem to improve the systemic control for overall survival, with distant metastases still occurring in 25–35% of patients [6].

The metastatic pattern in rectal cancer differs from that of colon originated disease. The venous drainage of the distal rectum through the iliac veins bypasses the portal system, and the iliac lymph nodes – a possible route for lymphatic cancer spread – are not removed during standard TME. Therefore, despite the liver being the most common site for distant metastasis, rectal cancer more frequently metastasizes to the lungs, nervous system, and bone, whereas peritoneal spreading is more common in colon cancer [7, 8]. Furthermore, differences in the carcinogenesis of rectal tumors have been recognized. Physical activity, lower body weight, and aspirin show a protective effect in colon cancer but not in rectal cancer [2]. Familial adenomatous polyposis causes cancers predominantly in the distal colon and rectum. However, most sporadic and Lynch syndrome‐associated tumors with microsatellite instability (MSI) are generally hypermutated and characterized by a strong host immune reaction as a response to the high expression of neoantigens, and they arise usually in the proximal colon. In addition, BRAF^V600E^ mutations are rare in rectal cancer [2, 9].

The host immune response has a well‐established role in constraining cancer. High quantities of tumor‐infiltrating CD3+ and CD8+ lymphocytes are associated with an improved prognosis in several cancer types [10], and this observation has led to numerous grading systems in an attempt to predict cancer behavior. Most notably, the Immunoscore®, derived from the CD3+ and CD8+ lymphocyte densities from the tumor invasive margin and center, has proven to be a strong prognostic marker in colon cancer [11]. In addition, lymphoid aggregates, referred to as Crohn's‐like lymphoid reaction (CLR) in CRC, play an important part in orchestrating the antitumoral reaction. CLR develops first from CD4+ T‐cell and antigen‐presenting dendritic cell clusters, later including B cells and follicular dendritic cells, which through maturation form organized tertiary lymphoid structures with active germinal centers. CLR is more common in MSI tumors and related to the high amount of tumor infiltrating lymphocytes, as tertiary lymphoid structures enhance and sustain the antitumoral immune reaction by providing a local site for the tumor antigen presenting for dendritic cells, which lead to the activation, proliferation, and differentiation of T and B cells [12, 13]. However, it is unclear how preoperative radiotherapy in rectal cancer modifies the antitumoral immune response and associated cancer demeanor.

The aim of this study was to evaluate the histopathologic features of rectal cancer after preoperative SRT or CRT, as well as the prognostic impact of the host immune response indicated by CRL and CD3+ and CD8+ lymphocytes.

## MATERIALS AND METHODS

### Patients

The study population consisted of 346 rectal cancer patients, a part of a large cohort of 1479 CRC patients with surgical resection at Central Finland Central Hospital during 2000–2015 with recently reported age‐adjusted Charlson comorbidity index (CCI) and associated multimodal management with updated survival data [14]. Adequate tumor samples for immunohistochemical studies were available for 1343 patients, of which 983 patients without preoperative therapy were analyzed in our previous study [15]. Here, we focus on rectal cancer patients, of whom 95 had preoperative SRT and 97 had CRT. For comparison, we included 154 rectal cancer patients without preoperative treatments, referred to as the nRT group (no radiotherapy). Complete responses were excluded from the study. Radical surgery was distributed as R0 (clean specimen marginals, n = 298), R1 (tumor growth to less than 1 mm from specimen marginals, n = 16), and R2 (unresectable primary tumor or distant metastasis, n = 32). Histological tumor parameters; CLR density (according to Väyrynen et al. [16]); lymphovacular invasion (LVI); tumor regression grade (TRG, according to Rödel et al. [17]); mucinous, stromal, and necrotic component; stroma maturity (according to Ueno et al. [18]); differentiation (according to the WHO criteria); and budding (according to the International Tumor Budding Consensus Conference [19]) were evaluated by a study pathologist (JPV) from hematoxylin and eosin (H&E)‐stained whole slide samples. Stroma maturity was defined as: (i) mature with fine and elongated collagen fibers stratified into multiple layers; (ii) intermediate with keloid‐like collagen intermingled with mature fibers; and (iii) immature consisting of myxoid stroma without mature fibers [18].

### Immunohistochemical analyses

Formalin‐fixed paraffin‐embedded (FFPE) tumor samples were used to prepare tissue microarray (TMA) blocks with a TMA Master II tissue microarrayer (3D Histech Ltd., Budapest, Hungary) containing two 1 mm diameter cores from representative areas of both the tumor center and the invasive margin. The blocks were then cut to 3.5 μm‐thick sections and immunohistochemistry for CD3+ and CD8+ T cells were performed by a BOND‐III automated IHC stainer (Leica Biosystems, Buffalo Grove, IL, USA) with monoclonal antibodies and protocols, as described by Elomaa et al. [15] Immunohistochemical screening for DNA mismatch repair (MMR) deficiency with MLH1, MSH2, MSH6, and PMS2 expressions and for BRAF^V600E^ mutation status was performed according to Seppälä et al. [20].

CD3 and CD8 immunohistochemistry was analyzed by supervised machine learning built with the open‐source bioimage analysis software QuPath [21] utilizing previously validated algorithms [15, 22]. The T‐cell density score (DS) was calculated from the densities of CD3^+^ cells in the tumor center, CD3^+^ cells in the invasive margin, CD8^+^ cells in the tumor center, and CD8^+^ cells in the invasive margin, which were converted to percentiles (0–100) according to the principles of Immunoscore [11]. DS was determined by calculating the mean of the four percentiles and categorizing it into three groups: low (0–25), intermediate (>25–70) and high (>70–100) [15]. In our previous study, we introduced the T‐cell proximity score (PS) as a measurement of tumor cell‐T‐cell co‐localization, which was associated with longer cancer‐specific survival independent of T‐cell densities. PS was calculated based on the G‐cross (G_Tumor:immune cell_) function values at a 20‐μm radius (evaluating the likelihood of any tumor cell in the sample having at least one immune cell of the specified type within 20 μm radius), converted to percentiles and categorized into three groups (0–25, >25–70, and >70–100) [15].

**Fig. 1:**
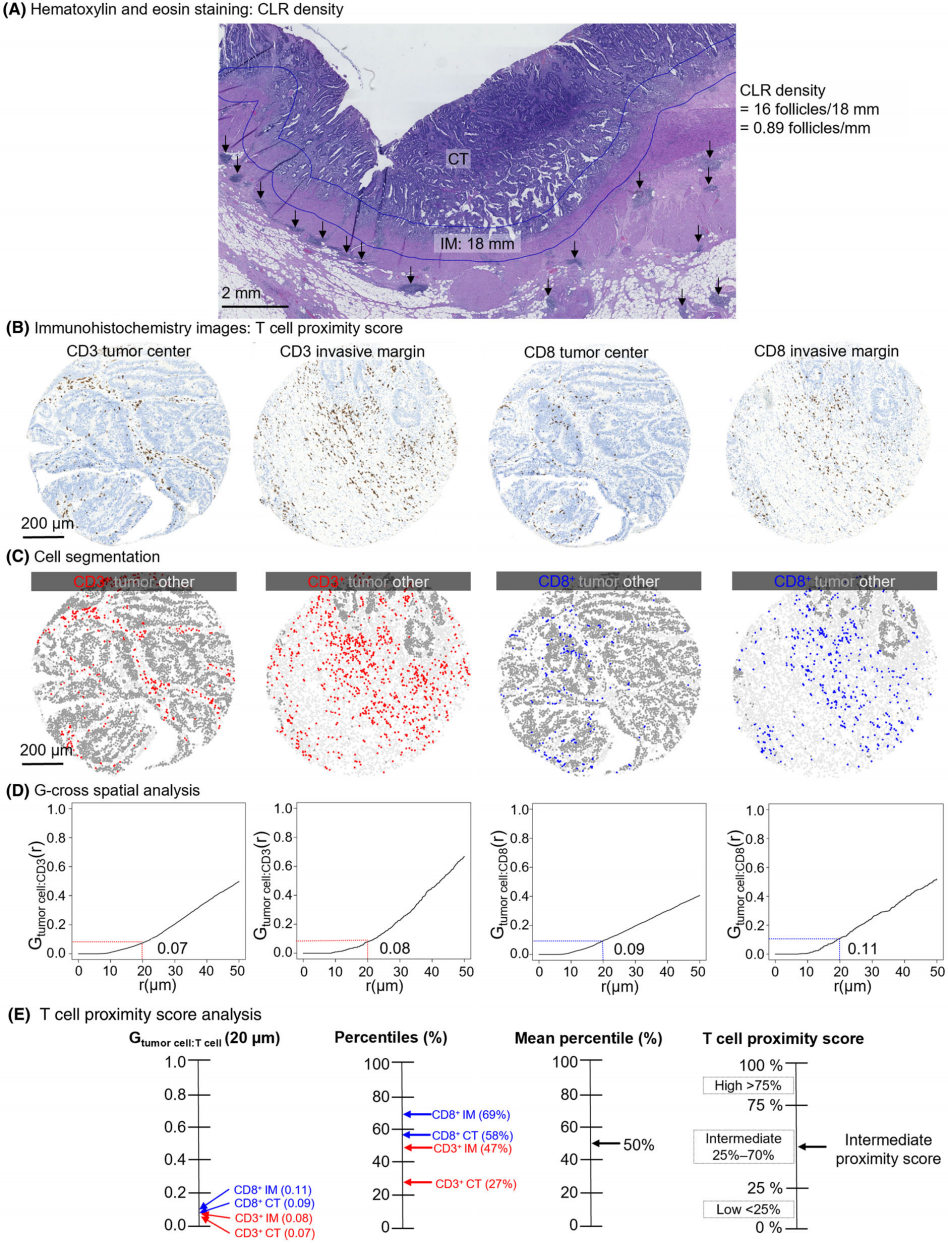
Crohn's like reaction density and T‐cell proximity score analysis. (A) Analysis of Crohn's like reaction density from a hematoxylin‐ and eosin‐stained whole‐slide image. (B) Examples of CD3 and CD8 immunohistochemistry images from the tumor center and invasive margin of a single tumor. (C) Corresponding phenotyping maps for T cells, tumor cells, and other cells. (D) G‐cross function curves, representing the likelihood of any tumor cell being co‐located with at least one T cell within radius r. (E) T‐cell proximity score calculation chart. G‐cross function values at r = 20 μm are converted into percentiles and, according to the mean of the four percentile values, the tumor is given a T‐cell proximity score.

CLR density, indicating the number of CLR follicles divided by the length of the analyzed invasive front, has been identified as a significant prognostic marker in CRC [16]. We selected a cutoff value of 0.25 follicles/mm obtained from a receiver operating characteristic (ROC) curve drawn in relation to disease‐specific mortality. Examples of CLR density and T‐cell proximity score analysis are shown in Fig. 1. CLR density, describing the local guidance of the host immune response, and PS, describing direct T cell‐to‐tumor cell interaction, were combined to form a more comprehensive parameter of the tumor immune environment, here referred to as the immune grade (IG). PS0 (0–25%), PS1 (>25–70%), and PS2 (<70–100%) were increased one category higher if the CLR density exceeded 0.25 follicles/mm, thus forming a four‐step scale of IG.

### Statistical analysis

Categorical data were compared using Pearson's chi‐square test. The Spearman correlation coefficient was used to determine correlations between immune cell infiltration and different treatment strategies. The Kaplan–Meier method was used to calculate disease‐specific survival (DSS) and overall survival (OS), and the log‐rank test was used to compare differences. A p‐value of <0.05 was considered statistically significant. Survival times were from the date of surgery to the time of death or to the end of the follow‐up. Survival analysis included only cancers with R0 resection, and cases with immediate postoperative deaths (n = 5) were excluded. Multivariable Cox proportional hazards regression models were used to analyze prognostic factors for DSS and OS. Statistical analyses were performed using IBM SPSS Statistics (version 27.0; SPSS Inc., Chicago, IL, USA).

## RESULTS

### Clinicopathological features

The median age of the patients was 69 (interquartile range, IQR 61–77) with a slight overrepresentation of the male gender (63%). The median follow‐up time after surgery was 6.9 years (IQR 3.1–10.5).

Clinicopathological variables in the different treatment groups are shown in Table 1. There was no statistically significant difference between the treatment groups according to age or sex. However, a higher CCI predicted exclusion from preoperative treatments (p = 0.009). When considering tumor‐associated parameters, there were no significant differences between treatment groups in TNM stage distribution, surgical radicality, tumor size, tumor budding, LVI, mucinous component, stroma maturity, or occurrence of distant metastases during follow‐up. Increased proportions of tumor necrosis and intratumoral stroma as well as poorer tumor differentiation was seen after SRT and CRT (p < 0.001).

**Table 1:** Clinicopathological variables in the different treatment groups

	nRT	SRT	CRT	All	p
	N of total 154 (% of column)	N of total 95 (% of column)	N of total 97 (% of column)	N of total 346 (% of column)	
Age					
<65	41 (27)	40 (42)	41 (42)	122 (35)	0.053
65–75	61 (40)	32 (34)	30 (34)	123 (36)	
>75	52 (34)	23 (24)	26 (24)	101 (29)	
Sex					
Male	92 (60)	61 (64)	66 (68)	219 (63)	0.404
Female	62 (40)	34 (36)	31 (32)	127 (37)	
CCI					
0–2	49 (32)	41 (44)	46 (47)	136 (40)	0.009
3	41 (27)	28 (30)	31 (32)	100 (29)	
≥4	63 (41)	25 (27)	20 (21)	108 (31)	
TNM stage					
I	53 (34)	23 (24)	20 (21)	96 (28)	0.121
II	40 (26)	34 (36)	29 (30)	103 (30)	
III	42 (27)	31 (33)	34 (35)	107 (31)	
IV	19 (12)	7 (7)	14 (14)	40 (12)	
Radicality of surgery					
R0	130 (84)	88 (93)	80 (83)	298 (86)	0.265
R1	7 (5)	3 (3)	6 (6)	16 (5)	
R2	17 (11)	4 (4)	11 (11)	32 (9)	
T‐cell density score					
0 (low)	26 (17)	50 (53)	23 (24)	99 (29)	<0.001
1 (intermediate)	93 (60)	43 (45)	50 (52)	186 (54)	
2 (high)	35 (23)	2 (2)	24 (25)	61 (18)	
T‐cell proximity score					
0 (low)	26 (17)	37 (39)	22 (23)	85 (24.5)	<0.001
1 (intermediate)	92 (60)	52 (55)	59 (61)	203 (58.5)	
2 (high)	36 (23)	6 (6)	16 (16)	58 (17)	
Immune grade					
0	11 (7)	21 (22)	19 (20)	51 (15)	<0.001
1	51 (33)	35 (37)	46 (47)	132 (38)	
2	61 (40)	35 (37)	30 (31)	126 (36)	
3	31 (20)	4 (4)	2 (2)	37 (11)	
CLR density					
Low	52 (34)	42 (44)	76 (78)	170 (49)	<0.001
High	102 (66)	53 (56)	21 (22)	176 (51)	
Tumor budding					
0–4/0.785 mm^2^	101 (66)	46 (48)	64 (66)	211 (61)	0.057
5–9/0.785 mm^2^	33 (21)	29 (31)	22 (23)	84 (24)	
≥10/0.785 mm^2^	20 (13)	20 (21)	11 (11)	51 (15)	
Lymphovascular invasion					
No	118 (77)	77 (81)	79 (81)	274 (79)	0.573
Yes	36 (23)	18 (19)	18 (19)	72 (21)	
Tumor grade					
1	46 (30)	11 (12)	13 (13)	70 (20)	<0.001
2	97 (63)	74 (78)	69 (71)	240 (69)	
3	11 (7)	10 (11)	15 (16)	36 (10)	
Mucinous tumor					
<50%	149 (97)	90 (95)	88 (91)	327 (95)	0.123
≥50%	5 (3)	5 (5)	9 (9)	19 (5)	
Stroma maturity					
1 (mature)	96 (62)	53 (56)	60 (62)	209 (60)	0.700
2 (intermediate)	22 (14)	20 (21)	15 (15)	57 (17)	
3 (immature)	36 (23)	22 (23)	22 (23)	80 (23)	
Tumor size					
0–40 mm	94 (63)	43 (46)	54 (56)	191 (57)	0.037
>40 mm	55 (37)	50 (54)	42 (44)	147 (43)	
Tumor necrosis					
<5%	32 (21)	5 (5)	12 (12)	49 (14)	<0.001
5–15%	106 (69)	59 (62)	66 (68)	231 (67)	
>15%	16 (10)	31 (33)	19 (20)	66 (19)	
Intratumoral stroma					
<50%	70 (46)	21 (22)	29 (30)	120 (35)	<0.001
≥50%	84 (55)	74 (78)	68 (70)	226 (65)	
Local recurrence					
No	116 (89)	85 (97)	71 (89)	272 (91)	0.108
Yes	14 (11)	3 (3)	9 (11)	26 (9)	
Distant metastasis					
No	96 (74)	70 (80)	51 (64)	217 (73)	0.067
Yes	34 (26)	18 (20)	29 (36)	81 (27)	

CCI, Charlson comorbidity index; CLR, Crohn's like reaction; CRT, chemoradiotherapy; nRT, no radiotherapy; SRT, short‐course radiotherapy.

CCI was determined without including the current colorectal cancer. CCI is missing from two patients. Tumor size is unknown in eight tumors. Local recurrence and occurrence of distant metastasis during follow‐up are evaluated from only R0 resected patients (n = 298).

T‐cell density score (DS) was lower after radiotherapy as DS0 was seen in 53% of the tumors after SRT and 24% after CRT vs 17% in the nRT group (p < 0.001). DS and T‐cell proximity score (PS) were distributed similarly in the nRT group. However, a slight shift toward higher PS was seen in SRT between DS and PS, but after CRT, the highest PS became less frequent. For CLR density, high densities were seen in 66% of the nRT group, in 56% after SRT, and only in 22% after CRT (p < 0.001). The immune grade (IG) was generally lower after SRT and CRT, as IG0 was seen in 7% and Grade 3 in 20% of the directly operated tumors compared to IG0 in 22% and IG3 in 4% after SRT and IG0 in 20% and IG3 in 2% after CRT (Table 1; p < 0.001).

The calculated median densities of CD3+ and CD8+ cells after different pretreatment modalities are shown in Fig. S1, and Table S1 presents corresponding correlation analyses. Overall, lower CD3+ lymphocyte counts were observed after both SRT and CRT compared to the nRT group. Although a markedly diminished amount of CD8+ cells were seen after SRT, there was no difference between the nRT and CRT groups.

### Immune contexture and association with histopathologic features

Histopathologic features according to T‐cell proximity score are shown in Table S2. Higher PS was associated with lower tumor grade and local tumor infiltration, less tumor spread to the lymph nodes, less distant metastasis, and overall lower TNM stage (p = 0.005, p < 0.001, p = 0.013, p = 0.007, and p < 0.001 respectively). Also, LVI and tumor budding were more common with a lower PS (p < 0.001 for both). Intratumoral stroma was more abundant in tumors with a low PS (p = 0.004), yet almost in all (84%) tumors with high PS, the intratumoral stroma was composed of mature collagen fibers, while in tumors with a low PS, the stroma more commonly (in 49%) consisted of myxoid stroma without mature collagen (p < 0.001). High CLR density was associated with higher PS (p = 0.001). Significant associations were not identified between the PS and tumor size, tumor regression grade, mucinous tumor type or the amount of intratumoral necrosis. Only four tumors were MMR deficient, none with a high PS. The BRAF^V600E^ mutation was found in nine tumors, three with PS0 and six with PS1. The occurrence of distant metastasis during the follow‐up was significantly more infrequent with the highest PS (p = 0.008).

Table 2 shows similar results with the immune grade. A higher IG was associated with lower TNM stage, less LVI and tumor budding, less intratumoral, yet more mature stroma and lower tumor grades (p = 0.001, p = 0.014, p < 0.001, p = 0.002, and p = 0.010, respectively). Local recurrences and distant metastasis were more common with a lower IG (p = 0.039 and p < 0.001, respectively).

**Table 2:** Histopathologic features according to the immune grade

	1	2	3	4	All	p
	N of total 51 (% of column)	N of total 132 (% of column)	N of total 126 (% of column)	N of total 37 (% of column)	N of total 346 (% of column)	
T						
1	1 (2)	8 (6)	12 (10)	6 (16)	27 (8)	0.001
2	8 (16)	27 (20)	37 (29)	18 (49)	90 (26)	
3	37 (72)	85 (64)	67 (53)	13 (35)	202 (58)	
4	5 (10)	12 (9)	10 (8)	0 (0)	27 (8)	
N						
0	26 (51)	69 (52)	91 (72)	26 (70)	212 (61)	0.013
1	17 (33)	39 (30)	21 (17)	9 (24)	86 (25)	
2	8 (16)	24 (18)	14 (11)	2 (5)	48 (14)	
M						
0	42 (82)	114 (86)	113 (90)	37 (100)	306 (88)	0.059
1	9 (18)	18 (14)	13 (10)	0 (0)	40 (12)	
TNM stage						
1	8 (16)	26 (20)	44 (35)	18 (49)	96 (28)	0.001
2	16 (31)	38 (29)	41 (33)	8 (22)	103 (30)	
3	18 (35)	50 (38)	28 (22)	11 (30)	107 (31)	
4	9 (18)	18 (14)	13 (10)	0 (0)	40 (12)	
Radicality of surgery						
R0	44 (86)	107 (81)	110 (87)	37 (0)	298 (86)	0.165
R1	2 (4)	8 (6)	6 (5)	0 (0)	16 (5)	
R2	5 (19)	17 (13)	10 (8)	0 (0)	32 (9)	
MMR status						
MMR proficient	51 (100)	130 (99)	124 (98)	37 (100)	342 (99)	0.709
MMR deficient	0 (0)	2 (1)	2 (2)	0 (0)	4 (1)	
BRAF						
Wild type	50 (98)	127 (96)	123 (98)	37 (100)	337 (97)	0.609
Mutation	1 (2)	5 (4)	3 (2)	0 (0)	9 (3)	
TRG						
Fibrosis <25%	26 (65)	43 (55)	51 (79)	5 (83)	125 (66)	0.078
Fibrosis 25–50%	9 (23)	25 (32)	8 (12)	0 (0)	42 (22)	
Fibrosis >50%	5 (13)	10 (13)	6 (9)	1 (17)	22 (12)	
LVI						
No	37 (73)	97 (73)	105 (83)	35 (95)	274 (79)	0.014
Yes	14 (27)	35 (27)	21 (17)	2 (5)	72 (21)	
Tumor budding						
0–4/0.785 mm^2^	23 (45)	68 (52)	86 (68)	34 (92)	211 (61)	<0.001
5–9/0.785 mm^2^	16 (31)	44 (33)	21 (17)	3 (8)	84 (24)	
≥10/0.785 mm^2^	12 (24)	20 (15)	19 (15)	0 (0)	51 (15)	
Tumor size						
≤40 mm	27 (54)	57 (44)	48 (39)	15 (44)	147 (43)	0.332
>40 mm	23 (46)	73 (56)	76 (61)	19 (56)	191 (57)	
Mucinous tumor						
0–49%	48 (94)	123 (93)	119 (94)	37 (100)	327 (94)	0.455
50–100%	3 (6)	9 (7)	7 (6)	0 (0)	19 (6)	
Tumor necrosis						
<5%	4 (8)	18 (14)	21 (17)	6 (16)	49 (14)	0.164
5–15%	30 (59)	92 (70)	84 (67)	25 (68)	231 (67)	
>15%	17 (33)	22 (17)	21 (17)	6 (16)	66 (19)	
Stroma maturity						
0 (mature)	19 (37)	70 (53)	86 (68)	34 (92)	209 (60)	<0.001
1 (intermediate)	5 (10)	32 (24)	18 (14)	2 (5)	57 (17)	
2 (immature)	27 (53)	30 (23)	22 (18)	1 (3)	80 (23)	
Intratumoral stroma						
<50%	10 (20)	37 (28)	55 (44)	18 (49)	120 (35)	0.002
≥50%	41 (80)	95 (72)	71 (56)	19 (51)	226 (65)	
Tumor grade						
1	6 (12)	18 (14)	33 (26)	13 (35)	70 (20)	0.010
2	37 (72)	97 (73)	83 (66)	23 (62)	240 (69)	
3	8 (16)	17 (13)	10 (8)	1 (3)	36 (10)	
Local recurrence						
No	39 (89)	92 (86)	105 (95)	36 (97)	272 (91)	0.039
Yes	5 (11)	15 (14)	5 (5)	1 (3)	26 (9)	
Distant metastasis						
No	28 (64)	65 (61)	91 (83)	33 (89)	217 (73)	<0.001
Yes	16 (36)	42 (39)	19 (17)	4 (11)	81 (27)	

LVI, lymphovascular invasion; MMR, mismatch repair; TRG, tumor regression grade.

Tumor regression grade according to Rödel includes only patients treated preoperatively with radiotherapy (N = 189). Tumor size is unknown in eight tumors. Local recurrence and occurrence of distant metastasis during follow‐up are evaluated from only R0 resected patients (n = 298).

### Univariable survival analysis

In the whole study population, CRT was associated with worse long‐term survival (10‐year DSS was 62% for CRT, 85% for SRT, and 77% for nRT, p = 0.006). For meaningful case numbers in the further univariable survival analysis, the SRT and CRT groups were combined as the preoperative RT group (pRT). Kaplan–Meier survival analyses with clinicopathological variables for the nRT and pRT groups are shown in Table 3. As expected, a higher CCI was prognostic for a worse 5‐year OS in both (p = 0.003 for the nRT group and p < 0.001 for the pRT group). A high TNM stage was significantly associated with a worse 5‐year DSS and OS in the nRT group (p = 0.005 and p = 0.008, respectively) and with a worse DSS in the pRT group (p = 0.004). Higher tumor budding was related to a worse DSS and OS in the nRT group (p < 0.001 and p = 0.008, respectively), but statistically significant differences in the pRT group were not identified (even when observing SRT and CRT separately). LVI was clearly associated with a higher disease‐specific mortality in both treatment groups (p < 0.001 for the nRT group and p = 0.007, for the pRT group). Additionally, the 5‐year OS was worse if LVI was identified, but significantly only within the nRT group (p = 0.044). Tumor grade did not have statistical significance in any of the group analyses (Table 3).

**Table 3:** Survival according to clinicopathological variables in the different treatment groups

	Five‐year disease‐specific survival						Five‐year overall survival					
	No RT (N = 128)			Preoperative RT (N = 166)			No RT (N = 128)			Preoperative RT (N = 166)		
	N	%	p	N	%	p	N	%	p	N	%	p
Sex												
Male	75	82	0.457	111	82	0.932	74	76	0.759	111	72	0.559
Female	54	82		55	88		54	74		55	75	
Charlson comorbidity index												
0–2	35	85	0.685	78	80	0.546	35	83	0.003	78	77	<0.001
3	40	81		49	85		40	75		49	76	
≥4	53	83		38	90		53	68		38	63	
TNM stage												
I	49	89	0.005	42	97	0.004	49	84	0.008	42	76	0.155
II	36	87		60	86		36	72		60	80	
III	39	75		58	76		39	66		58	67	
IV	4	50		6	40		4	50		6	33	
T‐cell density score												
0	19	61	0.050	59	81	0.986	19	58	0.386	59	73	0.434
1	74	87		84	84		74	76		84	73	
2	35	88		23	90		35	80		23	74	
T‐cell proximity score												
0	18	50	<0.001	47	81	0.466	18	39	0.002	47	70	0.919
1	76	84		99	83		76	77		99	74	
2	34	97		20	94		34	85		20	75	
Crohn's like reaction density												
Low	43	70	0.006	103	78	0.010	47	59	0.005	103	65	0.003
High	85	90		63	93		81	83		63	86	
Immune grade												
0	10	39	<0.001	33	75	0.019	10	30	0.001	33	64	0.126
1	37	74		70	79		37	67		70	70	
2	51	87		57	92		51	80		57	79	
3	30	100		6	100		30	87		6	100	
Low	47	68	0.001	103	78	0.003	47	59	0.005	103	68	0.027
High	81	92		63	93		81	83		63	81	
Tumor grade												
1	123	82	0.262	23	95	0.123	44	82	0.506	23	78	0.940
2	5	100		124	83		79	71		124	73	
3	0	n/a		19	79		5	60		19	68	
Tumor budding												
0–4/0.785 mm^2^	89	93	<0.001	98	88	0.274	89	82	0.008	98	78	0.361
5–9/0.785 mm^2^	25	64		45	78		25	56		45	67	
≥10/0.785 mm^2^	14	57		23	77		14	57		23	65	
Lymphovascular invasion												
No	102	88	<0.001	139	88	0.007	102	77	0.044	139	76	0.261
Yes	26	63		27	65		26	61		27	59	
Tumor regression grade												
1 (fibrosis <25%)				112	84	0.403				112	71	0.794
2 (fibrosis 25–50%)	Excluded			34	84		Excluded			34	71	
3 (fibrosis >50%)				19	84					19	84	

**Fig. 2:**
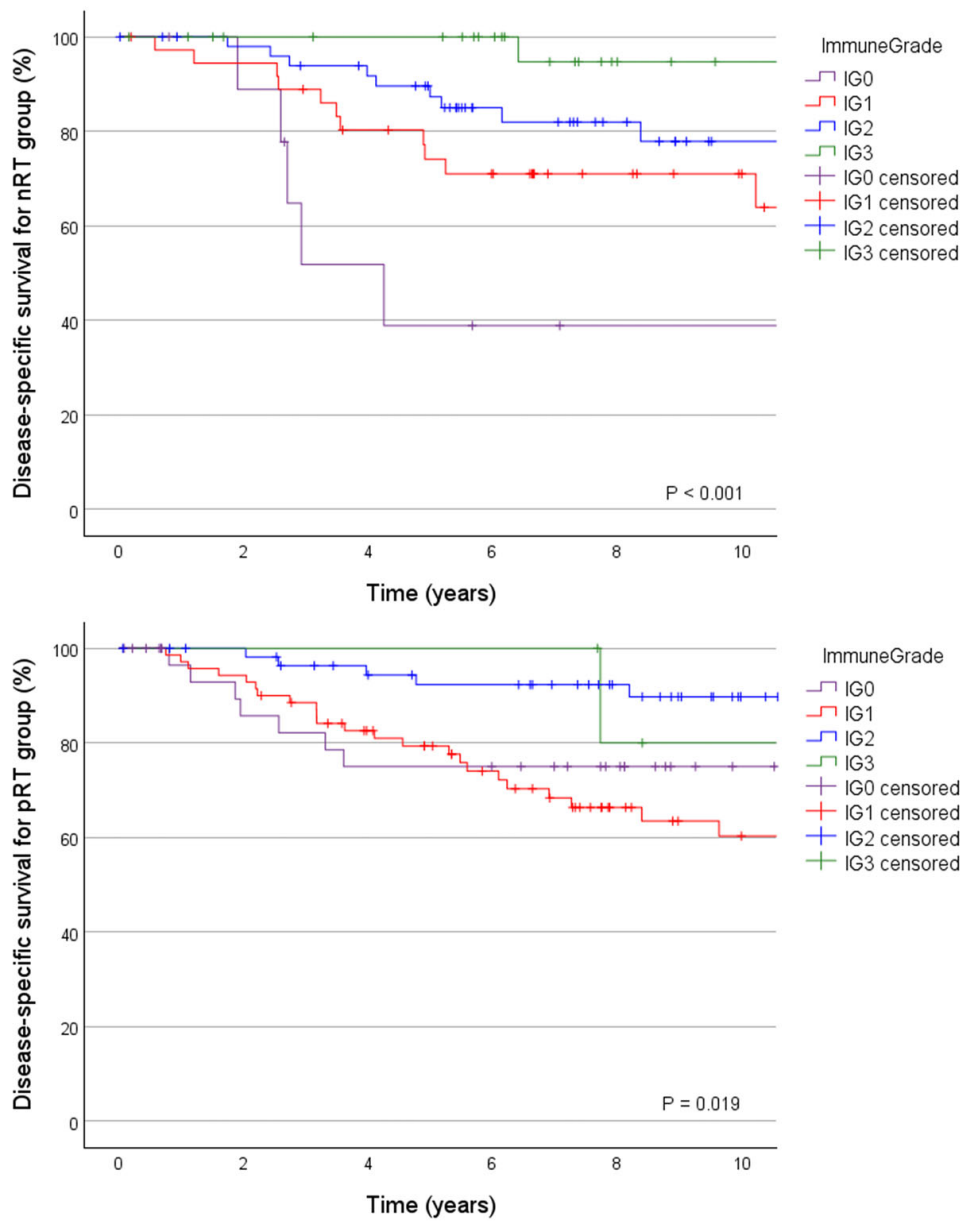
Disease‐specific survival according to immune grade for no radiotherapy (nRT) and preoperative treatment (pRT) groups.

When considering immunological parameters, the density score had no distinct impact on survival. The proximity score performed well in the nRT group (5‐year DSS for PS0 50% vs PS2 97%, p < 0.001, and 5‐year OS for PS0 39% vs PS2 85%, p = 0.001), but it did not adduce a clear survival benefit in the pRT group (Table 3). However, when evaluating treatment groups separately, clear trend for improved survival for high PS was observed in CRT group (10‐year DSS for high PS 92% vs 57% for combined PS0‐1, p = 0.072). In SRT group high PS was rare (only six tumors) and no significant differences were identified. High CLR density was associated with improved 5‐year survival in all analyses (DSS 70% vs 90%, p = 0.006, for the nRT group; 78% vs 93%, p = 0.010, for the pRT group; OS 59% vs 83%, p = 0.005, for the nRT group, and 65% vs 86%, p = 0.003, for the pRT group). Immune grade, that is, a combination of the PS and CRL density, improved the identification of the prognostic extremities especially in the nRT group (Table 3). Because of the similar outcomes (Fig. 2, Table 3), IG0 to IG1 and IG2 to IG3 were combined as low and high immune grades. In nRT group, the 5‐year DSS was for IG^low^ 68% and for IG^high^ 92% (p = 0.001) and the 5‐year OS for IG^low^ 59% and for IG^high^ 83% (p = 0.005). In pRT group, the 5‐year DSS for IG^low^ was 78% and for IG^High^ 93% (p = 0.003), and the 5‐year OS for IG^low^ was 68% and for IG^High^ 81% (p = 0.027).

### Multivariable survival analysis

Multivariable analysis with Cox proportional hazard model is shown in Table 4. The selected variables with significance in both treatment groups according to the Kaplan–Meier analysis included TNM stage, LVI, and IG together with the constant variables age‐adjusted CCI and sex. Additionally, the given preoperative therapy and TRG describing the achieved local effect at the time of the surgery were considered as key factors in standardizing the multivariable model.

**Table 4:** Multivariable analysis with Cox proportional hazard model

	Disease‐specific survival (DSS)		Overall survival (OS)	
	HR (95% CI)	p	HR (95% CI)	p
Charlson comorbidity index				
0–2	1	0.618	1	<0.001
3	1.36 (0.74–2.51)		1.85 (1.20–2.84)	
≥4	1.13 (0.59–2.15)		3.23 (2.14–4.89)	
Sex				
Male	1.14 (0.67–1.94)	0.630	1.15 (0.81–1.63)	0.427
Female	1		1	
TNM stage				
I	1	<0.001	1	<0.001
II	1.48 (0.69–3.15)		1.19 (0.77–1.83)	
III	1.80 (0.85–3.82)		1.32 (0.83–2.08)	
IV	14.67 (5.00–43.08)		5.63 (2.52–12.56)	
Immune grade				
Low	3.17 (1.76–5.71)	<0.001	1.96 (1.37–2.82)	<0.001
High	1		1	
Lymphovascular invasion				
No	1	<0.001	1	0.044
Yes	2.74 (1.58–4.78)		1.52 (1.01–2.29)	
Preoperative treatment				
None	0.57 (0.27–1.17)	0.004	0.51 (0.32–0.83)	0.010
Short‐course radiotherapy	0.25 (0.11–0.57)		0.51 (0.32–0.83)	
Chemoradiotherapy	1		1	
Tumor regression grade				
1 (fibrosis <25%)	1	0.073	1	0.253
2 (fibrosis 25–50%)	0.32 (0.12–0.85)		0.63 (0.35–1.16)	
3 (fibrosis >50%)	0.71 (0.27–1.88)		0.62 (0.29–1.34)	

Analysis includes only radically operated patients with postoperative deaths excluded (N = 294). Tumor regression grade (TRG) and Charlson comorbidity index (CCI) were missing from one patient. Reference categories were CCI 0–2, female sex, TNM stage 1, high immune grade, no lymphovascular invasion, preoperative chemoradiotherapy, and TRG 1. Tumors without preoperative therapy are included in the TRG 1 group (<25% fibrosis). Complete responses are excluded from the study.

Immune grade was an independent prognostic factor for survival [IG^low^ hazard ratio (HR) 3.17, 95% confidence interval (CI) 1.76–5.71, p < 0.001 for DSS and HR 1.96, 95% CI 1.37–2.82, p < 0.001 for OS]. High TNM stage was prognostic for worse survival (Stage IV HR 14.67, 95% CI 5.00–43.08, p < 0.001 for DSS and HR 5.63, 95% CI 2.52–12.56, p < 0.001 for OS). Also, LVI impaired both DSS (HR 2.74, 95% CI 1.58–4.78, p < 0.001) and OS (HR 1.52, 95% CI 1.01–2.29, p = 0.044). Compared to CRT, patients with SRT had improved DSS (HR 0.25, 95% CI 0.11–0.57, p = 0.004) and both nRT and SRT groups had better OS (HR 0.51, 95% CI 0.32–0.83 for both, p = 0.010). Those with higher CCI had worse OS (CCI ≥4 HR 3.23, 95% CI 2.14–4.89, p < 0.001).

## DISCUSSION

We aimed to better understand the changes induced by preoperative (chemo)radiotherapy to the immune contexture of rectal cancer. The prognostic effect of the tumor infiltrating lymphocytes is well documented in colon cancer. However, the rectum differs embryologically, anatomically, and functionally from the colon, and they cannot be compared unconditionally. In addition, the (chemo)radiotherapy often administered in rectal cancer might affect the quality of the tumor‐constraining immune response and subsequent survival benefit. Our results indicate that analysis of tumor immune contexture after preoperative treatments can still assist in predicting disease outcome in rectal cancer, as the combination of PS and CLR density (immune grade), was an independent prognostic factor for DSS and OS.

Radiotherapy is known to cause a wide spectrum of changes in the tumor microenvironment, some favorable and some harmful for the antitumoral struggle. Irradiation‐generated oxidative stressors cause damage to cancer cell DNA, followed by cell death, for example, through apoptosis, mitotic catastrophe, or cellular stress‐induced permanent cell cycle arrest [23]. The dying cells express damage‐associated molecular patterns (DAMP), which trigger the antitumoral immune response and immunogenic cell death. DAMPs enhance dendritic cell (DC) function with subsequent release of pro‐inflammatory cytokines that activate cytotoxic T lymphocytes [23, 24]. As DCs are central operators in triggering the antitumoral immune response, the prognostic effect of CLRs functioning as local platforms for DC antigen presentation is not surprising.

Tumor‐induced neovasculature is often more prone to irradiation damage because of the fast rate of vascular endothelial cell proliferation with an immature structure. The destruction of tumor vasculature causes hypoxia, which reduces the irradiation‐induced production of reactive oxygen species (ROS), the main mediator of tumor DNA damage. Hypoxia also supports cancer stem cell dormancy, further increasing the capability of cancer cells to endure radiotherapy [25, 26]. On the other hand, hypoxia, a common feature in aggressive rapidly growing tumors, leads to the upregulation of hypoxia‐inducible factors (HIF), supporting cancer cell survival. For example, HIFs increase the production of antioxidants capable of neutralizing ROS, suppress the immune functions of DCs, and recruit regulatory immune cells [26, 27] Also, the dosage of the radiotherapy is definitive to the impact on the tumor microenvironment. Although DCs are the most resistant immune cells, Tregs can endure larger doses than other lymphocytes. Thereby, higher doses may induce the selection of immunosuppressive immune T cells at the tumor site [28]. Fractioned low doses of radiotherapy promote the angiogenesis and transient increase in blood flow with tumor reoxygenation, allowing the increased infiltration of immune cells at the tumor site, but the antitumoral response is weaker. Large doses of irradiation cause severe vascular damage, inducing tumor necrosis and considerable extension of hypoxic areas, hence strongly promoting immunogenic cell death but also counteracting hypoxia‐driven immunosuppression [26, 29].

In the present study, we saw a significantly lower proportion of high densities of CLR in tumors after CRT compared to tumors with nRT and SRT. The longer waiting period before surgery and lower irradiation fraction of about 1.8 Gy might permit the shift toward regulatory immune response with the suppression of DC function and decreased recruitment of T‐helper cells responsible for the initial formation of lymphoid aggregates. Interestingly, the median CD3+ and CD8+ lymphocyte counts were lowest in the SRT group, and the remaining T‐cell infiltration seemed to have minor effect on cancer prognosis. SRT was followed by surgery within 10 days from the initial irradiation dose, but the radiation fraction was higher with 5 Gy given in the subsequent 5 days up to 25 Gy. Additionally, tumor necrosis was slightly more frequent after SRT compared to CRT. This reduced initial inflammatory reaction seen as a decrease in T lymphocyte and neutrophil infiltration after SRT compared to patients with surgery alone has been previously identified [30]. In fact, the timing of surgery may be of pivotal importance because the immune response and immunogenic cell death triggered by irradiation takes time to reach its peak. T‐cell infiltration is most abundant 7–14 days after the antigen encounter and then the short‐lived effector cells begin to wane, with only a small percentage surviving as memory T cells [31]. After SRT, the lymphocyte rate has been shown to recover within 7 days to the level seen in tumors without preoperative irradiation [30]. One possible explanation might be the destruction of the initially recruited local T cells, whereas their replacement might be slower because of tumor vasculature damage and the diminished migration of immune cells to the tumor site. Although irradiation can have strong immunostimulatory effects, a potent systemic antitumoral immune response is rarely seen due to the concomitantly stimulated immunosuppressive barrier. This realization has excited several research strategies combining irradiation with immunotherapies, generating promising results [27].

Previous literature on the effect of CRT on immune response in rectal cancer is conflicting [32]. A study of 130 rectal cancers with CRT compared to a cohort group of 30 non‐radiated cancers showed a decrease in both CD3+ and CD8+ lymphocyte levels, while the ratio of cytotoxic CD8+/granzyme B+ cells increased [33]. However, several studies with sequentially obtained specimens before and after chemoradiotherapy presented an increase in CD8+ lymphocytes [34, 35, 36, 37, 38] and in the expression of PD‐L1 overall [34], in tumor cells [39], or in immune cells [36]. Some studies presented a decrease in FOXP3 Treg cells [40], yet some reported unchanged densities [38]. Chiang et al. [41] presented an increase in overall PD‐L1, IFNy, and TGF‐β expression. We found that CRT was prognostic for worse DSS and OS compared to SRT, presumably reflecting the initially more advanced tumors selected for CRT. Still, the effects of activated immunosuppressive agents such as PD‐L1 or the diminished CLR presented here may have a critical role in cancer advancement. Therefore, it seems that the effect and the optimal timing of preoperative treatments in rectal cancer is far from solved.

Cancer‐associated fibroblasts (CAF) are associated with poor survival in many cancer types including CRC [42, 43]. In our study, we recognized significantly increased proportions of intratumoral stroma after CRT and especially after SRT, which may have immunosuppressive influences. Correspondingly, we could show that stronger immune responses were related to lower level of intratumoral stroma and mature collagen fibers, which is well established also in the literature [18]. Tumor budding, another important prognostic factor in CRC, had no significant impact on survival in either the SRT or CRT groups, even though there were no differences in identifying rates between groups. This conflicts with previous studies [44, 45]. It is possible that this phenomenon is disturbed by irradiation‐induced fibrosis or necrosis. However, this finding needs to be confirmed in larger studies.

BRAF mutation, especially the V600E subtype, is associated with worse survival in MSS colon cancer [20, 46]. However, in rectal cancer BRAF mutations are rare and non‐V600E mutations with a less dismal prognosis are seen more frequently [47]. Here, the BRAF^V600E^ mutation was identified in nine tumors (3%) of which seven were radically operated and only one was associated with MSI. The stage distribution was one Stage I, three Stage II, three Stage III, and two Stage IV diseases. Concordantly with colon cancer studies, disease outcome was significantly worse for patients with the BRAF^V600E^ mutation, as six (67%) of the nine patients with BRAF^V600E^‐mutated tumors had disease‐associated death (p < 0.001).

This study has some limitations. The data consist of patients from a timespan from 2000 to 2015, and improvements in multimodal management during this time may possibly have affected the survival analysis [14]. However, all the patients were treated at a single center, thus minimizing the variation in treatment policies. The number of patients in the different treatment groups is relatively small. The lymphocytes were calculated from TMA samples and may not represent the precise antitumoral immune response of the whole tumor, a matter discussed in our previous study [15]. However, the tumor characteristics were otherwise evaluated from whole‐slide H&E samples, providing a more comprehensive conception.

## CONCLUSION

In conclusion, overall lymphocyte infiltration is diminished after SRT, suggesting that surgery straight after irradiation may not be optimal in the sense of immune activation. However, as CLR seems scarcer after CRT, waiting too long might also weaken the durable antitumoral immune response. Proximity score (depicting T cell‐to‐tumor cell interaction) and CLR densities (depicting local guidance for immune response) are prognostic for DSS and OS in rectal cancer without preoperative treatments, and they emphasize that not only the number of infiltrating lymphocytes but also the location, clustering, and spatial interactions of the immune cells contribute to the prognostic evaluation. Proximity score and CLR density can be easily combined into an enhanced prognostic score called the immune grade. In the irradiated tumors, CLR density was the most significant immune factor for OS. Importantly the prognostic impact of T‐cell infiltration on survival was insufficient alone but combination with CLR density improved the performance of both on DSS. The immune grade proved to be an independent prognostic factor for DSS and OS when adjusted with CCI, sex, TNM stage, LVI, preoperative treatment modality, and TRG.

## FUNDING

E‐VW was supported by the Finnish Medical Foundation, the Mary and Georg C. Ehrnrooth Foundation, Cancer Foundation Finland, and the Ida Montin Foundation. TTS was supported by Cancer Foundation Finland, the Jane and Aatos Erkko Foundation, the Emil Aaltonen Foundation, the Sigrid Juselius Foundation, the Relander Foundation, and iCAN Flagship of the Academy of Finland. J‐PM was supported by Cancer Foundation Finland, the Jane and Aatos Erkko Foundation, and Finnish State Research Funding. JPV was supported by Cancer Foundation Finland.

## CONFLICT OF INTEREST

TTS is the CEO and co‐owner of Healthfund Finland Oy and reports consultation fees from Boehringer Ingelheim Finland and Amgen. Otherwise, the authors declare no conflict of interests.

## ETHICS APPROVAL AND CONSENT TO PARTICIPATE

The study was conducted according to the guidelines of the Declaration of Helsinki and approved by the hospital administration and the ethics board (Dnro13U/2011, 1/2016, and 8/2020) and the National Supervisory Authority for Welfare and Health (Valvira). The need to obtain informed consent from the study patients was waived (Valvira Dnro 3916/06.01.03.01/2016).

## Supporting information


**Fig. S1.** Median densities of CD3+ and CD8+ immune cells in different treatment groups: no radiotherapy (nRT), short‐course radiotherapy (SRT), and long‐course chemoradiotherapy (CRT).

**Table S1.** Correlation between lymphocyte counts and treatment groups.

**Table S2.** Histopathologic features according to the Proximity score.

## Data Availability

The datasets generated and/or analyzed during this study are not publicly available due to Finnish laws of privacy protection. The sharing of data will require approval from relevant ethics committees and/or biobanks. Further information including the procedures to obtain and access data of Finnish Biobanks are described at https://finbb.fi/en/fingenious‐service.
