# Supplementary figures and images for: The impact of preoperative treatments on the immune environment of rectal cancer
Source: APMIS. 2024 Sep 10;132(12):1046–60. doi: 10.1111/apm.13467 (PMC11582340; doi:10.1111/apm.13467)

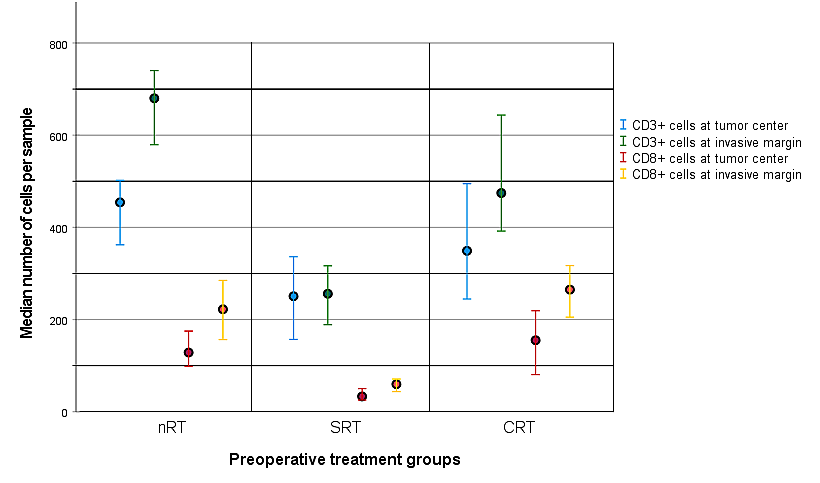

Supplement: Supplementary file 1 — Fig. S1. Median densities of CD3+ and CD8+ immune cells in different treatment groups: no radiotherapy (nRT), short‐course radiotherapy (SRT), and long‐course chemoradiotherapy (CRT). [file APM-132-1046-s002.tif]
