# Supplementary material for: The impact of preoperative treatments on the immune environment of rectal cancer
Source: APMIS. 2024 Sep 10;132(12):1046–60. doi: 10.1111/apm.13467 (PMC11582340; doi:10.1111/apm.13467)
Supplement: Supplementary file 2 — Table S1. Correlation between lymphocyte counts and treatment groups. [file APM-132-1046-s003.docx]

**Supplementary table 1. Correlation between lymphocyte counts and treatment groups**

|  | nRT vs. SRT | | nRT vs. CRT | |
| --- | --- | --- | --- | --- |
|  | Correlation coeffient | P | Correlation coeffient | P |
| CD3 tumor center | -0.307 | <0.001 | -0.073 | 0.238 |
| CD3 invasive margin | -0.438 | <0.001 | -0.130 | 0.038 |
| CD8 tumor center | -0.435 | <0.001 | 0.016 | 0.801 |
| CD8 invasive margin | -0.468 | <0.001 | 0.070 | 0.266 |
| Abbreviations: nRT: no radiotherapy; SRT: short-course radiotherapy; CRT: chemoradiotherapy. | | | | |
