# Supplementary material for: The impact of preoperative treatments on the immune environment of rectal cancer
Source: APMIS. 2024 Sep 10;132(12):1046–60. doi: 10.1111/apm.13467 (PMC11582340; doi:10.1111/apm.13467)
Supplement: Supplementary file 3 — Table S2. Histopathologic features according to the Proximity score. [file APM-132-1046-s001.docx]

**Supplementary table 2. Histopathologic features according to the Proximity score**

|  | **0**  N of total 85  (% of column) | **1**  N of total 203  (% of column) | **2**  N of total 58  (% of column) | **All**  N of total 346  (% of column) | **P** |
| --- | --- | --- | --- | --- | --- |
| **T:**  1  2  3  4 | 2 (2)  12 (14)  61 (72)  10 (12) | 17 (8)  49 (24)  121 (60)  16 (8) | 8 (14)  29 (50)  20 (34)  1 (2) | 27 (8)  90 (26)  202 (58)  27 (8) | <0.001 |
| **N:**  0  1  2 | 40 (47)  28 (33)  17 (20) | 130 (64)  45 (22)  28 (14) | 42 (72)  13 (22)  3 (5) | 212 (61)  86 (25)  48 (14) | 0.013 |
| **M:**  0  1 | 68 (80)  17 (20) | 182 (90)  21 (10) | 56 (97)  2 (3) | 306 (88)  40 (12) | 0.007 |
| **TNM stage:**  I  II  III  IV | 13 (15)  23 (27)  32 (38)  17 (20) | 53 (26)  69 (34)  60 (30)  21 (10) | 30 (52)  11 (19)  15 (26)  2 (3) | 96 (28)  103 (30)  107 (31)  40 (12) | <0.001 |
| **Radicality of surgery:**  R0  R1  R2 | 66 (78)  5 (6)  14 (16) | 176 (87)  10 (5)  17 (8) | 56 (97)  1 (2)  1 (2) | 298 (86)  16 (5)  32 (9) | 0.023 |
| **MMR status:**  MMR proficient  MMR deficient | 84 (99)  1 (1) | 200 (99)  3 (1) | 58 (100)  0 (0) | 342 (99)  4 (1) | 0.650 |
| **BRAF:**  Wild type  Mutation | 82 (96)  3 (4) | 197 (97)  6 (3) | 58 (100)  0 (0) | 337 (97)  9 (3) | 0.379 |
| **TRG:**  1 (fibrosis <25%)  2 (fibrosis 25-50%)  3 (fibrosis >50%) | 40 (71)  10 (18)  6 (11) | 73 (66)  27 (24)  11 (10) | 12 (54)  5 (23)  5 (23) | 125 (66)  42 (22)  22 (12) | 0.401 |
| **LVI:**  No  Yes | 56 (66)  29 (34) | 165 (81)  38 (19) | 53 (91)  5 (9) | 274 (79)  72 (21) | <0.001 |
| **Tumor budding:**  0-4/0.785 mm2  5-9/0.785 mm2  ≥10/0.785 mm2 | 32 (38)  33 (39)  20 (23) | 129 (63)  44 (22)  30 (15) | 50 (86)  7 (12)  1 (2) | 211 (61)  84 (24)  51 (15) | <0.001 |
| **Tumor size:**  ≤ 40mm  > 40mm | 45 (54)  39 (46) | 79 (40)  120 (60) | 23 (42)  32 (58) | 147 (43)  191 (57) | 0.095 |
| **Mucinous tumor:**  0-49 %  50-100 % | 77 (91)  8 (9) | 193 (95)  10 (5) | 57 (98)  1 (2) | 327 (95)  19 (5) | 0.121 |
| **Tumor necrosis:**  < 5 %  5-15 %  > 15 % | 8 (9)  54 (64)  23 (27) | 30 (15)  140 (69)  33 (16) | 11 (19)  37 (64)  10 (17) | 49 (14)  231 (67)  66 (19) | 0.165 |
| **Stroma maturity:**  0 (mature)  1 (intermediate)  2 (immature) | 33 (39)  10 (12)  42 (49) | 127 (63)  43 (21)  33 (16) | 49 (84)  4 (7)  5 (9) | 209 (60)  57 (17)  80 (23) | <0.001 |
| **Intratumoral stroma:**  < 50 %  ≥ 50 % | 17 (20)  68 (80) | 79 (39)  124 (61) | 24 (41)  34 (59) | 120 (35)  226 (65) | 0.004 |
| **TGP:**  Pushing  Infiltrative | 38 (45)  47 (55) | 139 (68)  64 (32) | 49 (84)  9 (16) | 226 (65)  120 (35) | <0.001 |
| **Tumor grade:**  1  2  3 | 9 (10.5)  60 (70.5)  16 (19) | 44 (22)  142 (70)  17 (8) | 17 (29)  38 (66)  3 (5) | 70 (20)  240 (69)  36 (10) | 0.005 |
| **CLR density**:  Low  High | 51 (60)  34 (40) | 98 (48)  105 (52) | 21 (36)  37 (64) | 170 (49)  176 (51) | 0.019 |
| **Local recurrence**  No  Yes | 58 (88)  8 (12) | 159 (90)  17 (10) | 55 (98)  1 (2) | 272 (91)  26 (9) | 0.104 |
| **Distant metastasis**  No  Yes | 44 (67)  22 (33) | 123 (70)  53 (30) | 50 (89)  6 (11) | 217 (73)  81 (27) | 0.008 |
| Abrevations: MMR, mismatch repair; TRG, tumor regression grade; LVI, lymphovascular invasion; TGP, tumor growth pattern; CLR, Crohn's like reaction.  Tumor regression grade according to Rödel includes only patients treated preoperatively with radiotherapy (N=189).  Tumor size is unknown in eight tumors. Local recurrence and occurrence of distant metastasis during follow up are evaluated from only R0 resected patients (n=298). | | | | | |
